# Effect of White Kidney Bean Flour on the Rheological Properties and Starch Digestion Characteristics of Noodle Dough

**DOI:** 10.3390/foods11223680

**Published:** 2022-11-17

**Authors:** Jiahui Han, Linjiang Pang, Linxin Bao, Xiafang Ye, Guoquan Lu

**Affiliations:** 1 Food and Health College, Zhejiang Agriculture and Forestry University, Hangzhou 311300, China; 2 Modern Agriculture College, Zhejiang Agriculture and Forestry University, Hangzhou 311300, China

**Keywords:** white kidney bean flour, noodle, rheological property, digestive property, dough microstructure

## Abstract

The aim of this study was to investigate the effect of adding white kidney bean flour on the quality of noodles. We selected four different proportions of white kidney bean flour (10–40%) in wheat flour to make the noodles, after which the noodles were analysed for their physical and chemical properties. The statistical method of correlation analysis was used in this study. The results showed that the noodles’ sensory and textural characteristics significantly improved after adding white kidney bean flour (*p* < 0.05). Compared with the control, the noodles’ surface with white kidney bean flour was denser and smoother. Moreover, microstructural observations indicated that the noodles with white kidney bean flour showed a more continuous protein network. The in vitro digestion results showed that the addition of white kidney bean flour reduced the digestibility of the noodles. Low addition of the flour (10–20%) improved the quality of the noodles, whereas high amounts (30–40%) showed the opposite effect. In this study, the optimal amount of white kidney bean powder was found to be 20%.

## 1. Introduction

Noodles are extremely popular in Asia and Europe because of their high nutritional value. To improve the processing properties of noodles, some researchers have added various flavouring substances to ordinary flour to make noodles. Many researchers have studied noodle formulations to improve the culinary properties of noodles. Some scholars added glycine and chickpea additives to wheat noodles and found that the addition of glycine at low concentrations effectively protected the gluten network structures of the noodles [1]. Some studies have reported that the formation of pores can shorten the optimal cooking time. Phosphate can cross-dense microstructures in noodles and improve water retention [2]. The addition of egg whites and oats to wheat noodles improves the quality of noodles through protein aggregation [3].

White kidney beans are pulses that are highly nutritious, containing rich protein, amino acid, fat, carotene, vitamins, polysaccharides, and a variety of trace elements [4]. The saponins, urinalysis, and a variety of globulins contained in white kidney beans can improve the immune ability of the human body, enhance the resistance to disease, inhibit the development of tumour cells, and also have a good effect on patients with liver coma [5]. White kidney bean contains highly active glycosidase hydrolases, which can hinder starch hydrolysis, reduce glucose absorption, and reduce lipogenesis. It has been found that white kidney bean extract has many special functions, such as anti-obesity, anti-diabetes, and blood glucose control effects [4].

Thus far, researchers have focused on the use of white kidney bean extract. Wang S 2021 found that adding white kidney bean extract into yogurt as a regulator has beneficial prebiotic metabolic properties [6]. Ma, Y., 2018, found that adding white kidney bean extract to different types of porridge can reduce the GI value of porridge [7]. However, the extent of white kidney bean use may be underestimated. For all the above mentioned developing a functional food using white kidney bean could be of great interest.

Replacing a proportion of wheat flour with white kidney bean flour for preparing noodles can improve the nutritional composition of the noodles and increase their nutritional value, but it may have negative consequences for the eating quality and processing characteristics.

In this study, we proposed an investigation on the effect of different additions of white kidney bean flour on the cooking quality of noodles and explored the interaction between white kidney bean flour and wheat starch and its effect on starch digestion in terms of rheological properties, microstructure, and starch hydrolysis rate of noodles. The aim is to provide a theoretical basis for the application of white kidney bean flour in noodles.

## 2. Materials and Methods

### 2.1. Materials

The white kidney bean flour was produced by Shandong Pair and Multi-Mac Foods Ltd. (Shandong, China). High-gluten wheat flour was purchased from local supermarkets. Other chemical reagents used for the experiments, such as glutaraldehyde and ethanol, were analytically pure (China Pharmaceutical Group Shanghai Chemical Reagent Company, Shanghai, China).

### 2.2. The Preparation of Noodles

A flour mixture (100 g) was prepared by mixing white kidney bean flour with wheat flour in the following ratios: 10:90, 20:80, 30:70, and 40:60 with a stirrer (UR2836 Philips mixer, Hangzhou, China)equipped with a spiral blade. Then, 45 mL of distilled water was added, and the dough was kneaded with a mixer for 2 min and rested at 23 °C for 30 min for rheological measurements. A portion of the pasta dough sample was freeze-dried and ground for further analysis. The noodles (2.4 mm width, 2.0 mm thickness, 200 mm length) were made by pressing the dough three times with a roll gap (3.0 and 2.0 mm) by using a pasta machine (Ningbo Hantanis Machinery Co., Ltd., Ningbo, China). The cooking process which was carried out is detailed in Section 2.3.3. Another dough and cooked noodles were prepared using 100% wholemeal flour without the addition of white kidney bean flour and were used in examining the effects of adding white kidney bean flour on the characteristics of prepared dough and noodles. A portion of the freshly cooked pasta was cooled to room temperature and kept for texture characterisation (TPA) determination. The remaining samples were freeze-dried until further analysis. Noodle dough and cooked pasta without adding the white kidney bean flour were used as controls.

### 2.3. Characterisation

#### 2.3.1. Analysis of the Rheological Properties of the Dough

The dough was made from white kidney bean flour, wheat flour, and gluten according to the above recipe, then covered with cling film and left to rest at room temperature for 30 min before being rheologically determined using a rheometer (TA Instruments Waters, Newcastle, DE, USA). The dough was laid flat in the centre of the rheological sump, the upper plate was slowly moved down to a distance of 5 mm between the upper and lower plates, and the excess dough was scraped off with a scraper. The frequency scan measurement parameters were circular plate (60 mm); temperature (25 °C); stress (0.5%); frequency variation (0.01–100 Hz); and variations in energy storage modulus (G′), loss modulus (G″), and loss angle tangent (tan δ) with frequency [8].

#### 2.3.2. Colour Difference Analysis of the Dough

White kidney bean flour and wheat flour were made into pasta sheets (20 × 10 × 20 mm) according to a recipe, and their colours were determined using a colourimeter. Three different areas were randomly selected on the pasta sheets, and their l*, a*, and b* values were recorded [9]. Whiteness index (WI) and Browning index (BI) were calculated according to the following literature [10]. The numbers 1–5 represent 0%, 10%, 20%, 30%, and 40%, and *i* represents 10%, 20%, 30%, and 40%.

(1) $$\Delta E=\sqrt{{\left(a_{i}-a_{1}\right)}^{2}+{\left(l_{i}-l_{1}\right)}^{2}+{\left(b_{i}-b_{1}\right)}^{2}}$$

(2) $$\mathrm{WI}=100-\sqrt{{\left(100-l\right)}^{2}+b^{2}+a^{2}}$$

(3) $$\mathrm{BI}=\frac{\left[\left(x*0.31\right)*100\right]}{0.172}$$

(4) $$x=\frac{a+1.75l}{5.645l+a-0.3012b}$$

#### 2.3.3. Analysis of Steaming Characteristics

The cooking characteristics of noodles include breakage, water absorption, and cooking loss [11]. Twenty noodles were taken, and their weight was measured and recorded as m0. A total of 500 mL of water was added to the pot and brought to a boil, and then the noodles were cooked for 5 min (the minimum time required to squeeze the noodles between two slides until the white core disappeared). The cooked noodles were rinsed in 300 mL of deionised water for 30 s and drained for 2 min before being weighed. Cooking water was collected and diluted to 500 mL with deionised water. After the noodles were removed, the number of intact noodles (N) and the weight of the noodles after cooking (m1) were determined. Diluted cooking water (L) was collected and dried to a constant weight; the beaker was weighed and recorded as m2, and the weighing was recorded as m3. Considering the small number of 20 noodles, 50 samples were used for the test of broken strip rate, and the method was the same as described above.

(5) Breakage rate(%)=N/50∗100

(6) Water absorption(%)=(m1−m0)/ m0∗100

(7) Cooking loss(%)=(m3−m2)∗50/m0∗100

#### 2.3.4. Sensory Evaluation

Sensory evaluation was conducted according to the method of Arise, A.K 2022 with some modifications [12]. Thirty trained volunteers between the ages of 20 and 60 years were recruited. Five samples (control, spaghetti with added white kidney bean flour) were served and coded in a random order in a uniform tray and immediately presented individually to group members. Volunteers rinsed their mouths between samples using mineral water. The attributes tested were colour, appearance, palatability, viscoelasticity, smoothness, and food acceptability. Each sensory descriptor was graded 1, 2, 3, or 4, according to the mean of the ratings given by the panellists, representing excellent, good, pass, or fail, respectively. Sensory results were analysed by using a stacked bar chart. Notably, we completed this sensory assessment within 20 min to ensure objectivity, and this procedure was conducted in an isolated room with good lighting and natural ventilation.

#### 2.3.5. Texture Characterisation of Cooked Pasta (TPA)

The noodles were cooked to the optimum cooking time and immediately cooled in tap water before measuring the textural properties of cooked noodle samples using a TMS-PRO texture analyser. The cooked noodles were collected in a stainless-steel strainer and placed in plastic bags. Measurements were carried out at room temperature within 15 min of cooking. Three cooked noodles were placed on the carrier table of a mass spectrometer (Stable Micro System, Surrey, UK)and compressed with a P5/R probe. The test parameters were as follows: starting force of 0.2 N, strain rate of 60%, velocity of 60 mm/s, and compression interval of 2 s. Each set of experiments was repeated at least nine times for each test [13]. The term “gumminess” was used to refer to the stickiness index of cooked noodles.

#### 2.3.6. Scanning Electron Microscopy

Samples of raw and cooked noodles were frozen in liquid nitrogen for 20 min, then placed in a freeze dryer (GAMMA1-16LSC lyophilizer, Marin Christ, Germany) and freeze-dried for 24 h before the samples were removed [14]. The noodle sample was gently broken off, and a small portion of the sample with a flat cross-section was selected and fixed to the upper surface of the carrier table with a conductive adhesive. We placed the samples in a scanning electron microscope (Cressington Scientific Instruments, Watford, UK)at an accelerating voltage of 10 keV to observe the morphological characteristics of the noodles [15]. The magnification was 1000 times.

#### 2.3.7. X-ray Diffraction Analysis

The crystallographic properties of freeze-dried pasta samples were determined using an X-ray diffractometer (Bruker D8 Advance, Karlsruhe, Germany) according to the method described by Jia, F., 2019 [1]. The scanning parameters of the samples were 40 kV and 100 mA. The relative crystallinity was calculated using Origin 2018 software (Origin Inc., Baltimore, MD, USA).

#### 2.3.8. Fourier Transform Infrared Spectroscopy

A 3 mg sample of lyophilised pasta was ground in a mortar (GAMMA1-16LSC lyophilizer, Marin Christ, Germany)and dried. The flake samples were then scanned on a Fourier-transform (TENSOR27; Bruker Optics GmBH, Ettlingen, Germany) infrared spectrometer with 32 scans at a resolution of 4 cm^−1^ and a range of 400–4000 cm^−1^ [1].

#### 2.3.9. Starch Digestion Properties of Noodles

The cooked pasta sample (1.00 g) in a 100 mL conical flask was weighed, and 15 mL of sodium acetate buffer (pH 5.2) was added. Then, 5 mL of enzyme solution (150,000 U porcine pancreatin α-amylase, amyloglucosidase) was added. The resulting mixture was shaken at 37 °C in a water bath at 200 r/min for 2 h. At 0, 20, 40, 60, 80, 100, and 120 min, 0.5 mL of each hydrolysis solution was mixed with 4.5 mL of anhydrous ethanol [16,17]. The samples were stored at 4 °C. The reducing sugar content was determined using the 3,5-dinitrosalicylic acid method [18].

#### 2.3.10. Statistical Analysis

The TPA experiment was performed nine times for reproducibility, and all the remaining experiments were repeated three times. Data are reported as mean ± standard deviation. IBM SPSS Statistics 26 software (SPSS Inc., Chicago, IL, USA) was used to analyse the significant differences in colour difference, cooking loss rate, texture quality, and digestive characteristics of noodles. Duncan’s multiple comparison test was used to determine statistical differences between means (*p* < 0.05).

## 3. Results

### 3.1. The Effect of Adding White Kidney Bean Flour on the Rheological Properties of the Dough

**Figure 1:**
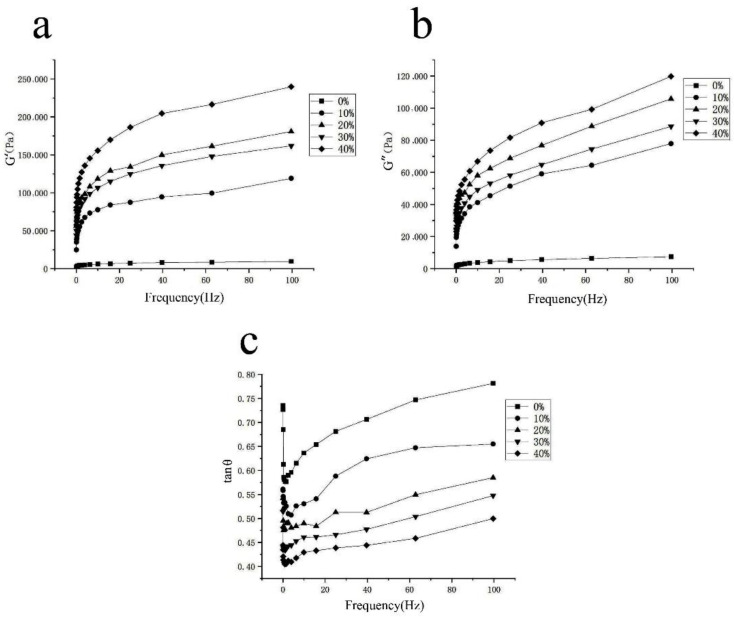
The effect of white kidney bean addition on (**a**) the storage modulus of noodle doughs; (**b**) the loss modulus of noodle doughs; and (**c**) tan δ of noodle doughs.

The effect of adding white kidney bean flour on the rheological properties of dough is shown in Figure 1. At a frequency range of 0.01–100 Hz, the dough’s energy storage and loss modulus showed a gradual increase after the white kidney bean flour content increased by 30%, and the loss angle tangent value decreased. The loss angle tangent value showed a general trend of decreasing and then increasing with increasing frequency. The dynamic moduli (G′ and G″) of the dough prepared with the white kidney bean flour–wheat composite flour were always higher than those of the all-wheat-flour-based dough, indicating that the replacement of wheat flour with white kidney bean flour increased the viscoelasticity of the dough samples. Moreover, the dynamic moduli (G′ and G″) of the dough samples with 20% white kidney bean flour were greater than those with 30% white kidney bean flour. The tangent of the loss angle of the dough was always less than 1, indicating that the energy storage modulus of the dough was always greater than the loss modulus and that the dough was mainly elastic rather than viscous [19]. The loss angle tangent value decreased with increasing white kidney bean flour content, indicating that the elasticity of the dough increased compared with the stickiness, and dough mobility gradually decreased. The loss angle tangent of the same dough tended to decrease and then increase with increasing frequency, indicating that the dough had high elasticity at low frequencies and the proportion of loss modulus increased at high frequencies. These effects result in an unstable structure that can be easily destroyed [20].

### 3.2. Analysis of Noodle Colour Difference and Cooking Quality

The effects of different additions of white kidney bean flour on colour difference in the noodles are shown in Table 1. After the addition of white kidney bean flour, the l* value of the noodles showed a decreasing trend, indicating that the whiteness of the noodles decreased. The gradual increase in the a* value of the noodles indicated that the colour of the noodles was closer to red. The b* value gradually increased, indicating that the yellowness of the noodles gradually increased [21]. With the increase in white kidney bean powder content, ∆E gradually decreased, indicating that the colour difference gradually decreased. BI gradually increased, indicating that the increase in kidney bean flour will lead to easy browning of the noodles. The whiteness index was highest with 10% white kidney bean powder, and 30% with whiteness above 20% or 40%. A small addition of white kidney bean powder showed better colour, with little difference in whiteness index between the addition of 20% and 40%.

As indicated in Table 2, the addition of white kidney bean flour had a definite effect on the cooking quality of the noodles. The cooking loss rate is a reflection of the loss of noodles in a broth during cooking and is the most important indicator of the cooking quality of noodles [22]. The quality of soup and cooking quality of noodles decrease with an increasing cooking loss rate. The possible reason is that white kidney bean flour dilutes gluten protein content and thereby affects the integrity of the gluten network structure, decreasing its ability to wrap contents [23]. With the increase in white kidney bean powder, the damage rate of noodles gradually increased. It may be that the water absorption rate of white kidney bean powder is higher than that of wheat flour, which leads to the difficulty of forming noodles under the same moisture conditions. As the amount of added white kidney bean flour increased, the increase in water absorption rate may have been due to the high water absorption capacity of white kidney bean flour, which was higher than that of wheat flour.

### 3.3. Sensory Characterisation of Noodles

**Figure 2:**
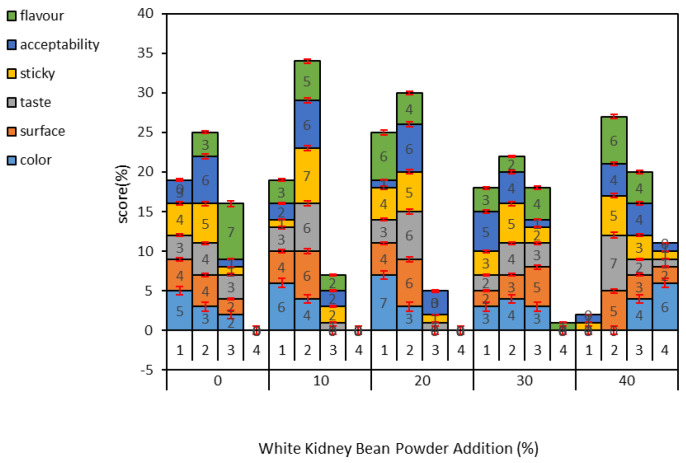
Sensory evaluation of noodles: 1–4 for represent excellent, good, pass, or fail, respectively; 0–40% represents white kidney bean flour addition.

As shown in Figure 2, the noodle colour score tended to decrease gradually after the addition of white kidney bean flour. When the amount of white kidney bean flour was 20%, the highest grade 1 score was obtained; when the amount of white kidney bean flour added was 40%, the lowest grade 1 score was obtained. On the one hand, the addition of a moderate amount of white kidney bean flour can make the gluten reticulation of the noodles tight, thus positively affecting the surface condition, palatability, viscoelasticity, and smoothness of the noodles and improving their organoleptic quality. On the other hand, SEM image results showed that adding white kidney bean flour in excess increased the porosity of dough and destroyed the structure of gluten network. This could lead to a reduction in the sensory quality of the noodles. Colour directly determines people’s perception of noodle quality. White kidney bean flour is yellowish and has a strong bean flavour. A small amount (10–20%) of white kidney bean flour mixed with wheat flour gives noodles a light-yellow colour and bean flavour. However, as the amount of white kidney bean flour added (30–40%) increases, the noodles become yellow and dull, and the bean flavour becomes extremely strong, affecting people’s desire to taste them. Noodles made with 20% added white kidney bean flour were rated as the most satisfactory by the study evaluation team. This is consistent with the highest cumulative grade 1 score of 20% in the figure.

### 3.4. Textural Characterisation of Noodles

The effects of different additions of white kidney bean flour on TPA in the noodles are shown in Table 3. With the addition of white kidney bean flour, the hardness of cooked noodles tended to increase. The hardness of the noodles is mainly determined by the deformed gluten mesh structure [16]. At a content of up to 30%, cohesiveness and gumminess showed a downward trend. Adhesiveness tended to increase and then decrease; at over 30%, all TPA features gradually increased. In general, the addition of a small amount of white kidney bean flour can improve the structural properties of noodles to a certain extent, whereas excessive white kidney bean powder will dilute the gluten protein in noodles, damage the network structure and reduce the quality of noodles.

### 3.5. Microstructural Analysis of Noodles

**Figure 3:**
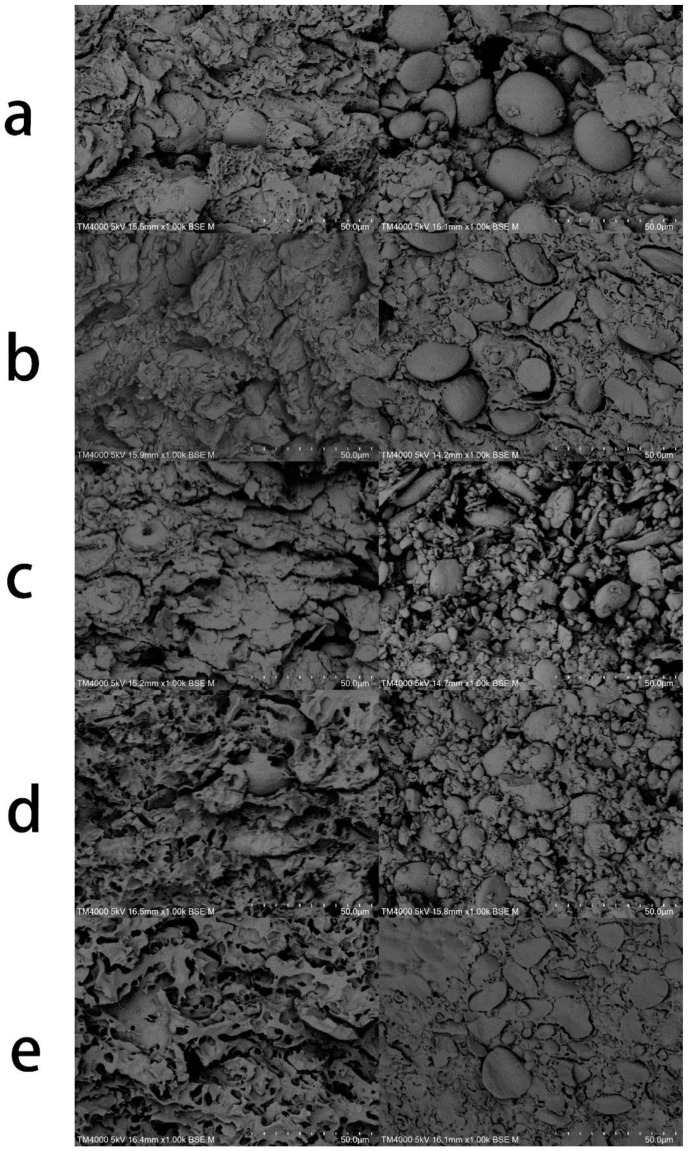
Effects of white kidney bean addition on the microstructure of dough by SEM (1000×). (**a**–**e**) White kidney bean additions of 0%, 10%, 20%, 30%, and 40%, respectively. The (**left**) side represents cooked noodles, and the (**right**) side represents raw noodles.

The microstructures of cooked noodles with different proportions of white kidney bean flour are shown in the left of Figure 3. The cross-section of cooked noodles without white kidney bean flour was a layered structure with many fine pores (Figure 3a—left). After the addition of white kidney bean flour (10–20%), many small gaps appeared in the cross-section of the cooked noodles (Figure 3b,c—left). Atter the addition of 30–40%, small gaps appeared, along with more big holes in the lamellar structure of gluten (Figure 3d,e—left). The number of small holes gradually increased with the amount of white kidney bean flour, and the diameters of the holes increased. The probable reasons were that with the addition of white kidney bean flour exerted a dilution effect on the gluten protein and that small starch particles dissolved in water and increased pore size. The larger pore size facilitated the entry of water into the centre of the pasta, promoting the pasting of starch in the pasta and reducing cooking time [24].

The microstructures of raw noodles with different additions of white kidney bean flour are shown in the right of Figure 3. The surface of raw noodles without white kidney bean flour was relatively rough and loose, and a large number of starch particles were free in the protein–gluten network structure and not wrapped (Figure 3a—right). However, after the addition of white kidney bean flour, the inclusions of starch molecules gradually increased; many starch molecules in the cooked pasta structure were well wrapped in the protein–gluten network structure; and the gap between starch molecules and gluten protein gradually decreased, forming a dense and smooth structure, thus changing the elasticity of the dough and increasing its hardness. These findings were consistent with the rheological and textural properties described above. The cross-section of cooked pasta with white kidney bean flour was flatter, starch molecules were well cut-off, and the control cross-section was mostly intact starch granules compared with those in the control.

### 3.6. X-ray Diffraction and Fourier Transform Infrared of Noodles

**Figure 4:**
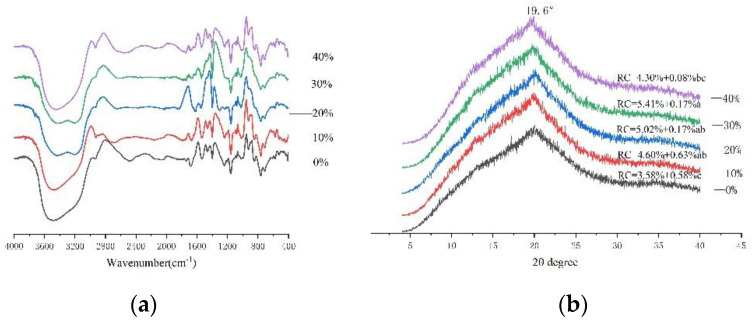
(**a**) FT-IR spectra of noodle doughs. (**b**) XRD spectra of noodle doughs. Different letters within the same group denote significant difference (*p* < 0.05).

As shown in Figure 4, the relative crystal (RC) obtained from the XRD spectra was higher than that of the pasta dough, suggesting that the addition of white kidney bean flour caused the dough to form a closely spaced array of crystals. The samples all had a peak at 19.6°, which was probably due to the combination of straight-chain starch and lipids forming a V-shaped crystal structure [1]. Furthermore, the intensity of the peaks gradually increased with white kidney bean flour content, indicating that the structure of the noodles was beginning to shift towards an amorphous structure [25]. The relative crystallinity of the noodles tended to increase and then decrease with increasing white kidney bean flour. In the noodle dough samples, the RC values increased with the addition of small amount of white kidney bean flour (10–30%) and then tended to decrease with the addition of a high amount of white kidney bean flour (30–40%). This change was consistent with the trend in G′ and G″ values and may have been due to physical competition between the continuous network of proteins and starch during the cooking process [26].

The FT-IR characteristic peaks of the noodles were shown at 3418, 3315, 3184, 2998, 2928, 2137, 1653, 1541, 1397, 1238, 1150, 1015, 926, 852, 568, and 526 cm^−1^, which are typical absorption peaks for these types of starch [27,28]. Exploring the molecular structures of starch granules in terms of short-range order is feasible [1]. The peaks at 3315 cm^−1^ and 1653 cm^−1^ were due to stretching vibrations of hydroxyl groups and stretching vibrations of CO, while the absorption peak at 1015 cm^−1^ was due to bending vibrations of C–OH and stretching vibrations of C–O in starch [27]. With the addition of white kidney bean flour, the peak at 1015 cm^−1^ slowly became lower, indicating the formation of hydrogen bonding interactions between white kidney bean flour and wheat starch. The broad absorption peak of 3000–3600 cm^−1^ was due to the interaction of bond energy and bond length in the hydrogen bond between the hydroxyl groups [29]. The peaks at 3418 cm^−1^ were due to hydrogen bonds formed by self-binding hydroxyl groups, and those at 3184 cm^−1^ were probably due to hydrogen bonding formed by the strong bonding of hydroxyl groups [10,30].

### 3.7. Analysis of the Digestive Properties of the Noodle Starch

**Figure 5:**
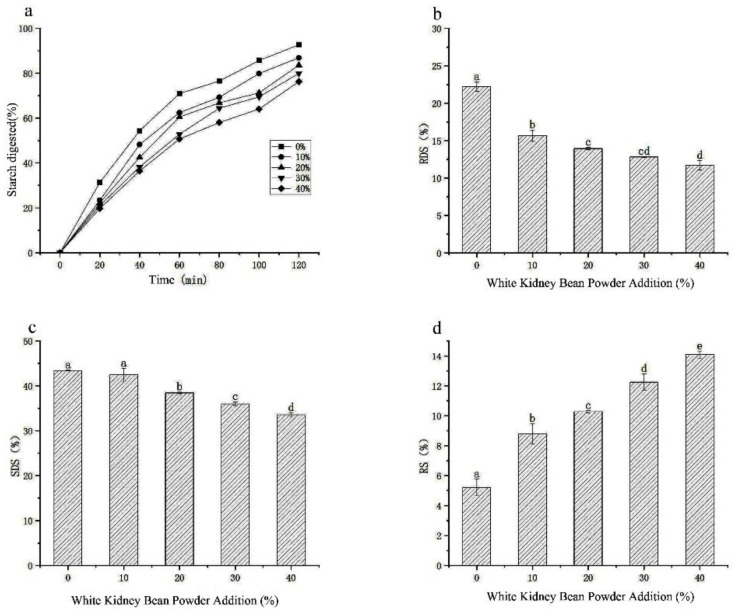
Digestion diagram of white kidney bean flour noodles with different additions: (**a**) starch hydrolysis rate; (**b**) fast digestible starch content (RDS represents fast digestible starch); (**c**) slowly digesting starch content (SDS represents slowly digestible starch); (**d)** resistant starch content. Duncan’s statistical methods were used. Different letters within the same group denote significant difference (*p* < 0.05).

In this experiment, the digestive characteristics of starch were analysed by determining starch digestibility. The results of the effect of different additions of white kidney bean flour on the starch digestibility of the noodles are shown in Figure 5. Figure 5a–d shows the significant differences in the starch hydrolysis rate, total starch content, RDS, SDS, and RS among the noodles with different additions of white kidney bean flour. As the amount of white kidney bean flour added increased, the starch hydrolysis rate decreased, total starch content decreased, fast-digesting and slow-digesting starch content decreased significantly, and resistant starch content increased significantly.

Reduction in the starch hydrolysis rate after the addition of white kidney bean flour could be attributed to two reasons. On the one hand, white kidney bean flour is rich in dietary fibre [31], which to a certain extent inhibits the hydrolysis of starch and absorption of glucose, reducing the production of fat. On the other hand, white kidney beans contain alpha-amylase inhibitors that prevent starch digestion by completely blocking access to the active site of the alpha-amylase enzyme [4]. The exact causes need to be further explored.

## 4. Conclusions

This study investigated the relationship between the addition of white kidney bean powder and the texture, sensory quality, and digestive properties of noodles. The contents of white kidney bean powder were 0%, 10%, 20%, 30%, and 40%. The results showed that different dosages of white kidney bean powder had significant effects on the rheological properties, in vitro digestion, and sensory evaluation of noodles (*p* < 0.05). Compared with the control, the contents of RDS and SDS were significantly decreased, and the contents of RS were significantly increased in the noodles supplemented with white kidney bean flour (*p* < 0.05). According to various studies, the sensory and texture characteristics of noodles were improved when the content of white kidney bean powder was 20%, the score of the sensory evaluation was the highest, and the rate of strip breaking was the lowest, which was the best choice. Therefore, white kidney bean powder can be used as a natural and safe auxiliary ingredient to improve the quality of noodles. The nutritional quality of white kidney bean noodles will be explored in further studies.

## Figures and Tables

**Table 1:** Effect of different additions of white kidney bean flour on the colour of the noodles.

Sample (%)	l*	a*	b*	∆E	WI	BI
0	70.92 ± 0.24 ^a^	3.03 ± 0.04 ^e^	19.23 ± 0.07 ^b^	-	-	-
10	70.80 ± 0.02 ^ab^	3.42 ± 0.05 ^d^	19.39 ± 0.32 ^b^	1.07 ± 0.27 ^a^	65.01 ± 0.17 ^a^	55.78 ± 0.01 ^d^
20	70.78 ± 0.01 ^ab^	3.87 ± 0.04 ^c^	19.81 ± 0.05 ^a^	1.04 ± 0.21 ^a^	64.10 ± 0.08 ^b^	55.92 ± 0.02 ^c^
30	70.54 ± 0.46 ^ab^	4.03 ± 0.06 ^b^	19.51 ± 0.08 ^b^	0.80 ± 0.30 ^a^	64.49 ± 0.03 ^ab^	56.05 ± 0.01 ^b^
40	70.31 ± 0.23 ^b^	4.25 ± 0.02 ^a^	20.04 ± 0.09 ^a^	0.79 ± 0.16 ^a^	64.16 ± 0.47 ^b^	56.11 ± 0.03 ^a^

Duncan’s statistical methods were used. Means with different small letter superscripts within the same column were significantly different at *p* < 0.05.

**Table 2:** Effect of different additions of white kidney bean flour on the cooking of the noodles.

Sample (%)	Cooking Loss (%)	Water Absorption (%)	Breakage Rate (%)
0	3.33 ± 0.56 ^c^	56.46 ± 0.24 ^c^	6.00 ± 0.02 ^a^
10	3.48 ± 0.23 ^bc^	58.57 ± 1.17 ^b^	0.00 ± 0.00 ^b^
20	3.76 ± 0.52 ^abc^	58.87 ± 0.30 ^b^	2.00 ± 0.00 ^bc^
30	4.25 ± 0.47 ^ab^	59.46 ± 0.84 ^b^	2.67 ± 0.01 ^b^
40	4.43 ± 0.37 ^a^	62.60 ± 0.80 ^a^	3.33 ± 0.01 ^b^

Duncan’s statistical methods were used. Means with different small letter superscripts within the same column were significantly different at *p* < 0.05.

**Table 3:** Effect of different additions of white kidney bean flour on the textural properties of the noodles.

Sample (%)	Hardness (N)	Adhesiveness (J)	Cohesiveness (Ratio)	Gumminess (N)	Chewiness (J)	Springiness (m)
0	9.35 ± 0.04 ^c^	0.19 ± 0.16 ^ab^	0.51 ± 0.03 ^a^	5.22 ± 1.20 ^a^	4.57 ± 1.33 ^ab^	0.87 ± 0.15 ^b^
10	10.50 ± 0.31 ^b^	0.17 ± 0.07 ^b^	0.47 ± 0.04 ^a^	4.66 ± 0.45 ^ab^	4.83 ± 0.80 ^a^	1.03 ± 0.12 ^a^
20	10.65 ± 0.18 ^b^	0.27 ± 0.06 ^a^	0.40 ± 0.00 ^b^	4.38 ± 0.42 ^bc^	4.53 ± 0.84 ^ab^	1.03 ± 0.14 ^a^
30	10.87 ± 0.09 ^b^	0.19 ± 0.03 ^ab^	0.38 ± 0.07 ^b^	3.96 ± 0.23 ^c^	3.87 ± 0.53 ^b^	0.97 ± 0.09 ^ab^
40	11.77 ± 0.13 ^a^	0.21 ± 0.09 ^ab^	0.40 ± 0.05 ^b^	4.23 ± 0.45 ^bc^	4.26 ± 0.76 ^ab^	1.00 ± 0.09 ^a^

Duncan’s statistical methods were used. Different letters within the same column denote significant difference (*p* < 0.05).

## Data Availability

The data presented in this study are available on request from the corresponding author.
